# Editorial: Molecular Mechanisms of Retinal Cell Degeneration and Regeneration

**DOI:** 10.3389/fcell.2021.667028

**Published:** 2021-03-05

**Authors:** Glenn P. Lobo, Manas R. Biswal, Altaf A. Kondkar

**Affiliations:** ^1^Department of Medicine, Medical University of South Carolina, Charleston, SC, United States; ^2^Department of Ophthalmology, Medical University of South Carolina, Charleston, SC, United States; ^3^Department of Pharmaceutical Sciences, Taneja College of Pharmacy, University of South Florida, Tampa, FL, United States; ^4^Department of Ophthalmology, College of Medicine, King Saud University, Riyadh, Saudi Arabia; ^5^Glaucoma Research Chair in Department of Ophthalmology, College of Medicine, King Saud University, Riyadh, Saudi Arabia

**Keywords:** retinal cell degeneration, RPE, cilia, AMD, BEST1

Retinal degeneration is characterized by the deterioration of highly differentiated cells within the neurosensory retina, such as photoreceptors (PR), retinal pigment epithelium (RPE), or the choroid. Retinal remodeling culminating with cell death and topological changes in the retina is the final common pathway in many retinal degenerative diseases (RDDs) that cause irreversible blindness. Identifying the molecular and cellular mechanisms responsible for these processes is crucial for early diagnosis and prevention of these pathological outcomes to thwart blindness remains the main goal in vision research and is quite challenging. This special issue of *Frontiers in Cell and Developmental Biology*, as a result of contributions from leading groups in this field has elegantly covered the different aspects of both clinical and basic research involved in the process of retinal degeneration associated with complex or Mendelian traits highlighting new mechanistic avenues.

Retinal ganglion cell (RGC) degeneration in glaucomatous optic neuropathy poses a significant challenge in treating and managing glaucoma. With increasing evidence for vascular dysfunction in glaucoma, Wareham and Calkins highlight the potential role of neurovascular dysfunction and raise pertinent questions related to its causal role in glaucoma. The review discusses the mechanisms of vascular dysfunction in neuronal degeneration due to breakdown in autoregulation and neurovascular coupling in the neuronal, vascular, and glial cells that comprise the neurovascular unit. The authors hypothesize roles of these cells in the breakdown, possibly via the gap junction proteins that may affect cell-cell communication, cause leaky blood-retinal-barriers, predispose RGCs to apoptosis, and contribute to glaucoma progression. The modulation of vascular regulation and neurovascular interactions can represent an interesting area of neuroprotective modality for glaucoma in the future.

The retina is one of the highest oxygen-consuming tissues in the human body, making it highly susceptible to oxidative damage by reactive oxygen species (Schmidt et al., 2003). Aerobic glycolysis accounts for up to 90% of the glucose needs of the PR. The review by Rajala describes the role of aerobic glycolysis in the survival and maintenance of the PR cells with a focus on the metabolic and non-metabolic functions of pyruvate kinase isoform M2 (PKM2) expressed in the retina. Besides, Sinha et al. have effectively highlighted the role and mechanisms by which riboflavins and their derivatives maintain retinal oxidative homeostasis. The review furthers our knowledge on the importance of this underappreciated vitamin to the retina beyond other antioxidants and vitamins such as A, D, and E. Notably, oxidative stress and choroidal vascular dysfunction are hypothesized to be critically involved in age-related macular degeneration (AMD) pathogenesis.

Tong and Wang highlight the mechanisms by which different oxidative stressors induce oxidative damage and may be responsible for AMD. The review discusses the mechanisms of RPE senescence and different modes of cell death in RPE cells induced by hydrogen peroxide (H_2_O_2_), 4-Hydroxynonenal (4-HNE), N-retinylidene-N-retinyl-ethanolamine (A2E), Alu RNA, amyloid β (Aβ), and sodium iodate (NaIO_3_). The authors point toward a need for future investigations to understand the nature of RPE cell degeneration and death in AMD, which may be beneficial for developing therapeutic strategies to treat AMD patients. Besides, the review article by Hadziahmetovic and Malek further elaborates on responses and underlying mechanisms of cells vulnerable to AMD, such as the PR, RPE, microglia, and choroidal epithelial cells to oxidative damage, dysregulated lipid peroxidation, inflammatory cytokines and choroidal vascular dysfunction in the pathogenesis of AMD. Anti-angiogenic vascular endothelial growth factor inhibitors are available for treating wet AMD. However, the common dry AMD is still untreatable. The review discusses the currently available and in-trial treatment options for wet and dry AMD.

Identification of causal variants in a complex polygenic disease like AMD is challenging. Genome-wide association study (GWAS) is a useful tool for analyzing complex diseases, as they provide glimpses of the molecular pathways that lie beneath the disease landscape. Nguyen et al. describe an integrated approach of using GWAS and expression quantitative trait loci studies to prioritize functional variants that may be more likely to have a causal role and how such approaches have been successful in identifying causal genes in AMD. Likewise, Dhirachaikulpanich et al. have, for the first time, using an integrated transcriptomic approach, reported differentially expressed (DE) transcripts associated with mixed AMD in post-mortem macular (764 genes) and non-macular (445 genes) human RPE/choroid microarray and RNASeq datasets (GSE135092 and GSE29801). Interestingly, protein-protein interaction network identified two central hub genes, *HDAC1* and *CDK1*, involved in the control of cell proliferation/differentiation processes. The study highlights a useful approach to integrate publicly available datasets to increase the power of detecting functional transcripts and pathways that may provide novel insights into disease mechanisms and form the basis of future investigations.

The RPE is responsible for the proper functioning of the photoreceptors and maintenance of the blood-retinal barrier. RPE dysfunction can result in progressive loss of PR survival and subsequent loss of vision. Damaged RPE cells lose pigment, proliferate, migrate and differentiate into different cell types but may not transdifferentiate into neural retinal cells. Instead, they may form fibroblast-like cells, which, in the wet form of AMD, are involved in developing a choroidal neovascular membrane. These phenotypic changes in RPE cells are commonly referred to as ‘epithelial-mesenchymal transition' (EMT) (Zhou et al., 2020). The interesting review by Zhou et al. describes the clinical and pre-clinical evidence of EMT in the RPE cells and their role in AMD, proliferative vitreoretinopathy and certain inherited forms of retinal degenerations plausibly via tight junctions and adherens junctions proteins and the unfolded protein response (UPR) pathway which is involved in the repair and removal of damaged or misfolded proteins. The authors propose a role of UPR pathway and provide evidence for the role of misfolded proteins in RPE dysfunction that may have pathological consequences.

Mutations in *BEST1* (Bestrophin 1) are responsible for different inherited retinopathies, including the autosomal dominant Best vitelliform macular dystrophy or Best disease (BD). A study by Bonilha et al. describes the retinal findings in two donors harboring heterozygous variants in the *BEST1* gene: c.886A>C (p.Asn296His) (in donor 1) or c.602T>C (p.Ile201Thr) (in donor 2) to suggest that bestrophin-1 localization is mutation-dependent and support the concept that different variants in the *BEST1* gene can result in substantially different phenotypes. In another inherited retinal disease like Bardet-Biedl syndrome (BBS), which is caused by mutations in the *BBS* gene, Song et al. using a *bbs2*^−/−^ deficient cone dominant zebrafish model has elegantly demonstrated how the loss of *bbs2* leads to impaired visual function in larval zebrafish and progressive photoreceptor cone degeneration in adults; and how acute injury stimulates Müller glia proliferation and modest regeneration of cones. As the authors suggest, the zebrafish *bbs2* deficient model may represent an ideal tool to investigate mechanisms involved in retinal degeneration diseases to promote regeneration.

Considering the increasing rate of visual impairment and blindness worldwide (from estimated 45 million blind in 1996, to projected 76 million in 2020), it is imperative to look for feasible therapeutic approaches to prevent them (Ackland et al., 2017). The recent success of gene therapy (Luxturna^TM^) for Retinitis Pigmentosa and Leber's Congenital Amaurosis caused by retinoid isomerohydrolase (RPE65) mutations have raised the hopes of similar ongoing trials in inherited forms of retinal diseases. The concept of deriving retinal cells from human embryonic stem cells and induced pluripotent stem cells or endogenous sources can offer a promising and exciting prospect for stem cell therapy in retinal degenerative diseases. The current status and challenges to make advancements toward therapeutics have been reviewed by Ikelle et al.

Overall, the 12 contributions that make up this special issue provide a broad overview of the molecular mechanisms involved in retinal cell degeneration and certain inherited forms of retinal diseases, recent advances and potential regeneration strategies that should be the major goal of investigators in this field. The quality of the reviews and research articles are excellent. Retinal degeneration is a vast field, and although many questions remain to be answered, exciting discoveries will continue to be made in this area in years to come.

## Author Contributions

GPL and AAK wrote the editorial. MRB read and edited the editorial. All authors contributed to the article and approved the submitted version.

## Conflict of Interest

The authors declare that the research was conducted in the absence of any commercial or financial relationships that could be construed as a potential conflict of interest.
